# Patterns of depressive symptoms among older adults living in long-term care homes in China: a latent class analysis

**DOI:** 10.3389/fpubh.2026.1861090

**Published:** 2026-07-01

**Authors:** Hongwei Qi, Sihan Dong, Shaoqing Ge, Yuting Song

**Affiliations:** 1 School of Nursing, Qingdao University, Qingdao, Shandong, China; 2 Qingdao University Affiliated Hospital, Qingdao, Shandong, China; 3 The University of Texas at Austin School of Nursing, Austin, TX, United States; 4 Faculty of Nursing, University of Alberta, Edmonton, AB, Canada; 5 Qingdao Municipal Key Laboratory for Smart Chronic Disease Management and Digital Rehabilitation Nursing, Qingdao, Shandong, China

**Keywords:** depressive symptoms, latent class analysis, long-term care homes, mental health, older adults

## Abstract

**Background:**

Depressive symptoms among long-term care (LTC) residents are heterogeneous. This study aimed to identify latent classes of depressive symptoms among older adults in Chinese LTC homes and examine their associations with social support.

**Methods:**

A cross-sectional study included 329 older adults from 11 LTC homes. Depressive symptoms and social support were measured using the PHQ-9 and Perceived Social Support Scale. Latent class analysis (LCA) identified symptom patterns, and multinomial logistic regression evaluated associations with social support.

**Results:**

LCA identified three clinically interpretable classes: low depressive symptoms (54.1%), somatic-dominant depressive symptoms (22.5%), and severe-spectrum depressive symptoms (23.4%). The severe-spectrum group exhibited the highest physical dependency, multimorbidity, and social isolation. Higher levels of perceived support from family and friends were associated with lower odds of belonging to the severe-spectrum group.

**Conclusion:**

Depressive symptoms in LTC residents can be described by clinically interpretable symptom patterns. Perceived social support may be relevant to depressive symptom differentiation in LTC residents and may inform psychosocial care in LTC settings.

## 1 Introduction

Depressive symptoms remain a significant and persistent challenge among older adults living in long-term care (LTC) homes. In China, depressive symptoms are reported among 37.5% of residents living in LTC homes (1), which is notably higher than the estimated 20.0% among older adults in community settings in China (2). LTC residents are particularly susceptible to emotional distress due to drastic changes in their living environment and diminished social support (3). Compounding this vulnerability is the fact that most LTC homes primarily prioritize basic daily living care, often lacking comprehensive psychological support services (4). Such widespread prevalence has serious consequences for older adults. Depressive symptoms not only deteriorate individuals’ physical health and quality of life but also heighten the risk of self-harm and suicide (5, 6). Beyond its clinical consequences, depression has significant economic implications, increasing healthcare resource consumption and resulting in productivity losses. In older adults, depressive symptoms are associated with increased health service utilization and higher healthcare costs (7). Consequently, depressive symptoms have emerged as a critical health priority requiring urgent attention and targeted intervention within the nursing facility service system (8).

Depressive symptoms are widely recognized as heterogeneous, with distinct symptom subtypes that may vary across cultural contexts (9, 10). A study of residents in U.S. LTC homes identified four symptom patterns: minimal symptoms, fatigue, depressed mood, and multiple symptoms (11). In contrast, a Spanish study categorized depressive symptoms in older adults into three subgroups: psychosomatic, melancholic, and anhedonic (12). Notably, differences in institutional models, staffing, and resident case-mix of LTC homes across countries should be considered when comparing findings across studies. Recent cross-cultural research suggests that cultural background may influence the expression and structure of depressive symptoms (13–15). Comparative research between China and the United States reveals differences in the subjective experience and reporting patterns of depression in the two countries (13). Broader cross-cultural analyses further indicate that the structure of depressive symptoms is not consistent across cultural groups (14). People in East-Asian cultures tend to use emotional regulation strategies, such as suppression and avoidance, more often than people in western cultures (16), which might affect how they express emotional distress. People from Asian cultures tend to exhibit more physical symptoms and fewer psychological symptoms than their counterparts in western countries, who more frequently exhibit emotional distress and psychological suffering (17, 18). Therefore, the patterns of depressive symptoms identified among older adults in other cultures may not generalize to those in China.

Existing research on the heterogeneity among older adults in China has primarily focused on the community-dwelling population (19), including older adults living alone (20), frail older adults (21), and older adults bearing family caregiving responsibilities (22). Among older adults living alone, subgroups with low, moderate, and high levels of depression were identified (20). For frail older adults, the study further identified four distinct subgroups characterized by different dominant features: loneliness-dominant, suicidal ideation-prominent, negative emotion-dominant, and high depression-high suicidal ideation (21). Similarly, among older adults raising grandchildren, three depression profiles (low, moderate, and high) were reported, and profile membership varied by gender, marital status, pension insurance, physical health, and life satisfaction (22). Little attention has been given to residents of LTC homes, who differ from community-dwelling older adults in their living environments and social support networks. Therefore, the patterns of depressive symptoms observed among community-dwelling older adults or in studies from other countries may not generalize to older adults living in LTC homes in China.

In this study, we employed latent class analysis (LCA) to identify patterns of depressive symptoms among LTC residents in China. Unlike traditional variable-centered approaches that focus on summed severity scores, LCA is a person-centered statistical method that identifies unobserved subgroups based on shared symptom patterns, allowing for the categorization of older adults in LTC homes into groups with similar presentations of depressive symptoms (23–25). To assess the clinical relevance of the identified patterns, we examined whether the profiles differed in perceived social support, a well-established psychosocial correlate of depressive symptoms in older adults and a particularly relevant factor in LTC settings (26, 27). Therefore, we aimed to identify latent profiles of depressive symptoms among older adults living in LTC homes in China and to examine whether the identified profiles differed in resident perceived social support.

## 2 Methods

### 2.1 Study design

We conducted a cross-sectional study. Data were collected using a structured questionnaire from July 2023 to June 2024.

### 2.2 Setting and participants

We used convenience sampling to recruit older adults from 11 LTC homes in Qingdao City, Shandong Province. Qingdao is a developed coastal city in eastern China with a population of approximately 9 million. We included LTC homes that provide integrated medical and social care. In addition to providing housing and assistance with daily living activities, these facilities were staffed with nurses and doctors who could provide routine health monitoring and basic medical care. Most residents in the facilities were older adults with chronic conditions and varying degrees of functional dependence and required ongoing assistance and daily support.

Eligible participants were aged ≥60 years, had lived in the LTC home for >3 months, and were able to communicate verbally or in writing and complete the interview. We excluded older adults who had severe mental illnesses or had vision, hearing, or cognitive impairments that interfered with communication.

### 2.3 Recruitment

Before data collection, researchers contacted LTC administrators, explained the study aims and significance, and obtained administrative approval. Care aides assisted in identifying and recruiting eligible residents. Before interviews, researchers explained the purpose of the study and provided instructions for completing the questionnaire to potential participants.

### 2.4 Measurement

#### 2.4.1 Variables for LCA

Depressive symptoms were measured using the 9-item Patient Health Questionnaire (PHQ-9) (28). PHQ-9 assesses symptoms over the preceding 2 weeks (29). The PHQ-9 is feasible for LTC residents and shows high specificity (29, 30). The scale has nine items scored 0 (not at all) to 3 (nearly every day), yielding total scores from 0 to 27. Higher scores indicate more severe depressive symptoms. Total scores of 0–4, 5–9, 10–14, and ≥15 correspond to none, mild, moderate, and severe depressive symptoms, respectively. The LCA was based on responses to the nine individual PHQ-9 items. The standard PHQ-9 total score cutoffs (0–4, 5–9, 10–14, and ≥15) were used only to describe the distribution of depressive symptom severity in the sample. Internal consistency was acceptable (Cronbach’s *α* = 0.777) in this study.

#### 2.4.2 Variables for assessing the clinical relevance of LCA-identified classes

To assess the clinical relevance of the identified classes, we examined whether class membership differed in perceived social support from family, friends, and a significant other (31, 32). Differences in perceived social support may reflect meaningful variation in psychological well-being and social functioning across classes (33). Social support was assessed using the Perceived Social Support Scale (PSSS) (34). This 12-item scale measures support from family, friends, and a significant other, yielding three distinct subscales. In the subsequent multinomial logistic regression analysis, the three subscales (support from family, friends, and a significant other) were included as independent variables to determine which specific aspects of social support differentiated the latent classes. Items are rated on a 7-point scale; subscale and total scores are sums, with higher values indicating greater perceived support. Higher total scores indicate greater perceived social support. Internal consistency was high (Cronbach’s *α* = 0.874) in this study.

### 2.5 Other covariates

Covariates included age, gender, education, marital status, body mass index (BMI), number of children, frequency of visits in the previous 6 months, pre-retirement occupation, number of comorbidities, self-rated health, length of stay, activities of daily living (ADL), frequency of participation in group activities, and type of residence. Table 1 summarizes all covariates.

**Table 1:** Summary of measures.

Variable	Description	Items	Scoring/categories
Variable for LCA (measured with LTC residents survey)			
Depressive symptoms	LTC residents reported the frequency of symptoms experienced over the past 2 weeks, covering both physical dimensions (such as sleep disturbances and fatigue) and cognitive-emotional dimensions (such as anhedonia and depressive mood).	9	Not at all, several days, more than half the days, nearly every day
Variables for assessing the clinical relevance of LCA-identified classes (measured with LTC residents survey)			
Support from family	LTC residents reported the extent of perceived support and understanding received from three distinct sources: family, friends, and a significant other.	4	Very strongly disagree, strongly disagree, mildly disagree, neutral, mildly agree, strongly agree, very strongly agree
Support from friends		4	
Support from a significant other		4	
LTC residents demographic characteristics (measured with LTC residents survey)			
Sex	LTC residents’ sex.	1	Male/female
Age	Chronological age (years).	1	64–74, 75–89, ≥90
Education level	Highest educational qualification attained.	1	Primary and below, middle school, high school, college and above
BMI	Body mass index (kg/m^2^)	1	Underweight, normal, overweight
Marital status	Current marital status.	1	Married, no spouse (widowed/divorced/unmarried)
Number of children	Total number of living children.	1	Categorical: 0, 1, 2, 3, ≥4
Frequency of visits in the previous 6 months	Frequency of visits to LTC residents over the past 6 months	1	Almost every day, at least once a week, at least once a month, at least once every 6 months
Occupation before retirement	Primary occupation held before retirement.	1	Party and government organization workers, workers in public organizations, enterprise personnel, free traders, laborers, farmers, soldiers
Number of comorbidities	Number of chronic conditions among LTC residents.	1	0–1 types, 2–3 types, ≥4
Self-rated health	The health status as perceived by LTC residents.	1	Very good, good, fair, poor, very poor
Length of stay	Duration of residence in the LTC home.	1	≥3 months<7 months, ≥7 months<12 months, 1–3 years, ≥3 years
Frequency of participation in group activities	Frequency of participation in group activities organized by the LTC homes.	1	Regular, occasionally, never
Type of residence	Living arrangement within the LTC home.	1	Living alone, living with spouse, living with other older people
ADL	Activities of daily living.	1	Normal, mildly dependent, moderately dependent

LCA, latent class analysis; LTC home, long term care home; ADL, activities of daily living.

### 2.6 Study size

Although some authors recommend samples >500 for LCA (12), no consensus standard exists. Previous LCA studies have included samples ranging from <100 to >500 participants (35, 36). Considering feasibility and drawing on prior studies, we set the sample size at 329.

### 2.7 Data collection procedure

Questionnaires were administered on site through one-to-one structured interviews. Before data collection, trained data collectors assessed the capacity of potential participants to provide informed consent using two open-ended questions adapted from the Evaluation to Sign Consent tool and used in our previous research: (1) “Please name two possible risks of participating in this study”; and (2) “What do you need to do to participate in this study?” (37, 38). Participants who provided reasonable answers to both questions were considered able to provide informed consent. Those who passed this screening and provided written informed consent were then offered a paper questionnaire. The data collectors explained how to complete the questionnaire using standardized instructions and assisted participants in completing the structured interview in person. Interviewers followed a standardized script, encouraged honest responses, and avoided leading language. Anonymity and confidentiality were maintained throughout data collection. Completed forms were collected on site and checked for completeness. The final analytic sample included 329 participants with complete data on all variables used in the analyses. Therefore, no missing-data imputation was performed, and all analyses were based on complete cases.

### 2.8 Statistical analysis

To explore the structure of depressive symptoms, we performed LCA, a probabilistic method that identifies unobserved subgroups from response patterns across multiple indicators (39). We used as indicators the 9 PHQ-9 items, each ranging from 0 (not at all) to 3 (nearly every day). LCA was preferred over latent profile analysis, which assumes continuous indicators. Factor mixture modelling was not selected because the purpose of this study was descriptive class enumeration of item-response patterns, and the sample size limited the stability of more highly parameterized models.

Analyses were conducted in R (version 4.5.0) using the poLCA package (40). For class enumeration, models with two to five classes were estimated. Each model was estimated with 300 random starts (nrep = 300) and a maximum of 5,000 iterations (maxiter = 5,000) to reduce the risk of local maximum solutions. Model fit was evaluated using log-likelihood (LL), Akaike information criterion (AIC), Bayesian information criterion (BIC), and entropy. BLRT and Lo–Mendell–Rubin tests were not reported because these likelihood-ratio tests are not directly implemented in the poLCA workflow used for this analysis.

Average posterior probabilities were calculated for each assigned class to assess classification quality. To further assess solution stability, an expanded random-start audit was conducted for the selected three-class model. Agreement between the audited maximum-likelihood solution and the exported class assignments was evaluated using the adjusted Rand index and best-matched classification agreement. Local independence was assessed descriptively using bivariate residuals (BVRs). Sensitivity to PHQ-9 coding was examined by re-estimating the three-class model after recoding items into binary indicators (0 = not at all; 1 = several days or more) and three-category indicators (0 = not at all; 1 = several days; 2 = more than half the days or nearly every day). Models with higher LL and lower AIC/BIC indicated a better fit–parsimony balance (40). Entropy ranges from 0 to 1; values >0.80 suggest adequate classification accuracy (40). We assessed model stability through sensitivity analyses by sequentially removing each PHQ-9 item and re-running the LCA to examine whether the overall class structure remained broadly similar.

After selecting the optimal model, participants were assigned to the most likely class based on posterior probabilities (41). We compared characteristics across classes using *χ*^2^ tests (categorical variables) and ANOVA (continuous variables). Fisher’s exact test was used when expected cell counts were <5.

To assess the clinical relevance of the identified classes, we used multinomial logistic regression to examine associations between depressive-symptom categories and social support from family, friends, and a significant other. Covariates were selected based on previous literature (26, 42, 43), clinical relevance, univariate between-class differences, and adequate cell counts for stable estimation (44). The final model adjusted for age, number of comorbidities, self-rated health, ADL, and frequency of participation in group activities, which were considered plausible confounders of the association between perceived social support and class membership. To reduce model instability, variables with sparse cells or limited conceptual relevance to the social support analysis were not included in the final multivariable model. Given the modest class sizes, interaction terms were not included in the primary model. Multicollinearity was assessed using variance inflation factors (VIFs), which ranged from 1.12 to 2.43 and indicated no serious collinearity among predictors.

Analyses were performed using SPSS 27.0 and R 4.5.0. Statistical significance was set at *p* < 0.05. We report odds ratios (ORs) with 95% confidence intervals (CIs).

## 3 Results

### 3.1 Participant characteristics

A total of 329 older adults participated in the study. The mean (SD) age was 82.92 (7.21) years, and 59.9% were female (*n* = 197). The mean (SD) depressive symptom score was 4.74 (4.23). For descriptive purposes, PHQ-9 total scores were categorized into four severity levels based on predefined cutoffs: none, mild, moderate, and severe. The corresponding proportions were 56.8% (*n* = 187), 30.1% (*n* = 99), 9.7% (*n* = 32), and 3.3% (*n* = 11).

### 3.2 Latent classes of depressive symptoms

We evaluated five LCA models, and the fit statistics are summarized in Table 2. Model selection was based on a comprehensive assessment of statistical fit, class size, entropy, and interpretability. Although the four-class model had a slightly lower AIC than the three-class model, the three-class model had a lower BIC. Because BIC imposes a stronger penalty for model complexity, it is often considered particularly informative for class enumeration. In addition, the four-class model yielded a class comprising only 5.2% of participants, suggesting limited stability and interpretive value. By contrast, the three-class solution produced more balanced class proportions (52.45, 24.25, and 23.31%), acceptable entropy (0.820), and clinically interpretable symptom patterns. Sensitivity analyses based on sequential removal of individual PHQ-9 items showed that the general three-class structure remained broadly similar, further supporting the robustness of the selected solution. Taken together, these considerations supported the selection of the three-class model as the most appropriate solution in this study.

**Table 2:** Latent class model fit indicators for depressive symptoms.

Model	LL	*G*-squared	AIC	BIC	Entropy	Class proportions
2-class model	−2475.048	1783.489	5060.095	5268.878	0.8642105	31.01/68.99
3-class model	−2419.898	1673.573	5005.797	5320.870	0.8199793	23.31/24.25/52.45
4-class model	−2383.829	1601.453	4989.658	5411.020	0.8598914	52.33/5.2/23.12/19.35
5-class model	−2358.133	1549.790	4994.267	5521.919	0.8938497	16.67/11.07/13.95/5/53.3

LL, Log Likelihood; AIC, Akaike information criterion; BIC, Bayesian information criterion.

The average posterior probabilities for the assigned severe-spectrum, somatic-dominant, and low depressive symptoms classes were 0.938, 0.881, and 0.940, respectively, indicating good classification quality. In an expanded random-start audit, the highest log-likelihood was −2419.898 and was replicated 47 times across 300 starts, suggesting that the selected solution was unlikely to represent a local maximum. The class assignments from this maximum-likelihood solution were highly consistent with the exported three-class classification (adjusted Rand index = 0.962; best-matched classification agreement = 98.8%). Local-independence diagnostics showed residual associations among several PHQ-9 item pairs; 16 of the 36 item pairs had BVRs >10, with the largest values observed for Item 6–Item 9 (BVR = 44.753), Item 3–Item 5 (BVR = 32.932), and Item 3–Item 4 (BVR = 26.628).

Sensitivity analyses partly supported the robustness of the three-class interpretation. The three-category coding solution showed good agreement with the original classification (best-matched agreement = 87.2%; adjusted Rand index = 0.718), whereas binary recoding was less stable (best-matched agreement = 73.3%; adjusted Rand index = 0.418). These findings suggest that the class structure was broadly retained when symptom-frequency information was partly preserved, but became more sensitive when the PHQ-9 items were substantially simplified.

**Figure 1:**
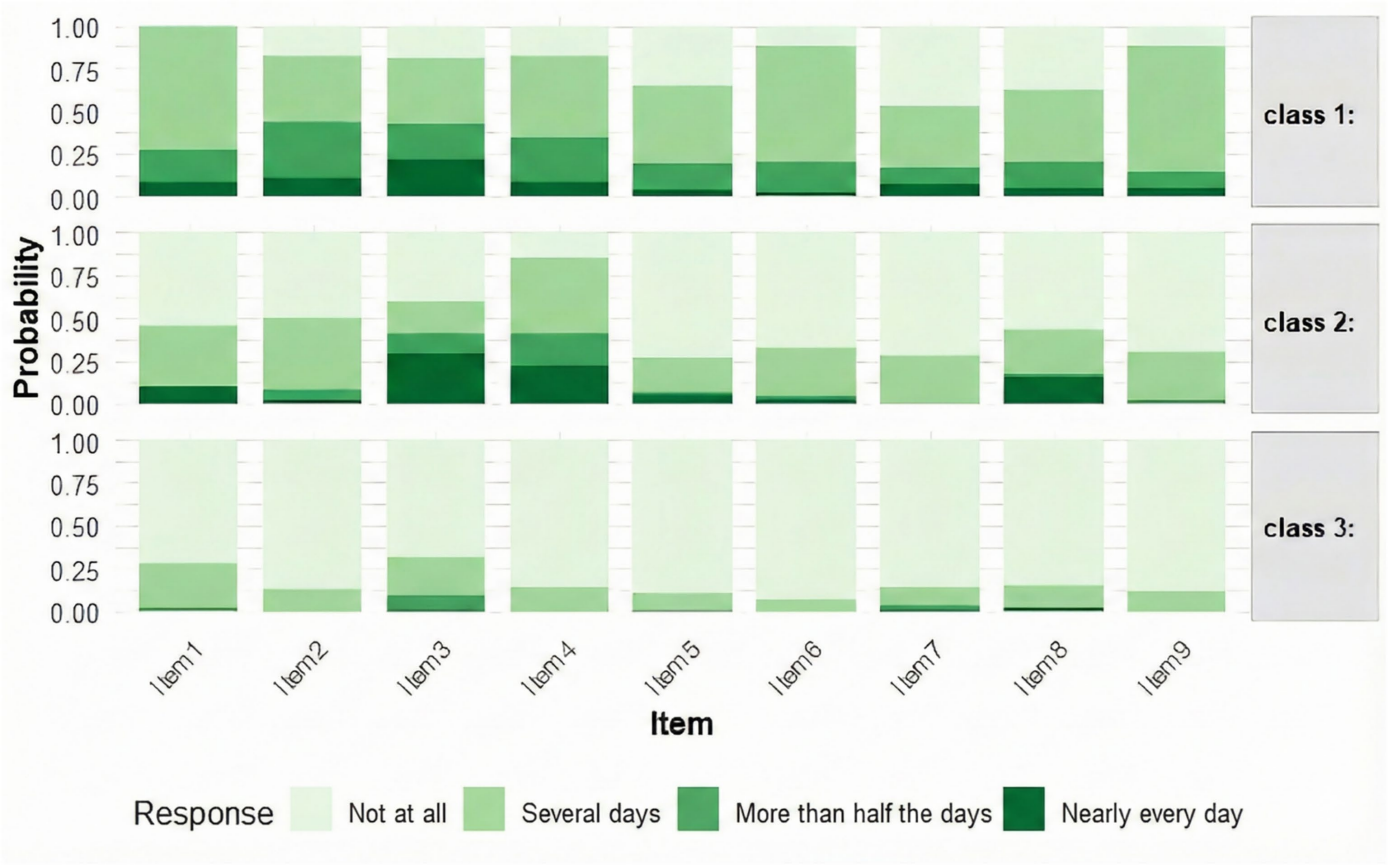
Probability of responding not at all, several days, more than half the days, nearly every day for each item of depressive symptom by identified classes of older adults. Item 1: anhedonia; Item 2: sadness; Item 3: sleep; Item 4: fatigue; Item 5: appetite; Item 6: worthlessness; Item 7: concentration; Item 8: movement; Item 9: suicidal ideation.

The conditional probability plot (Figure 1) shows, for each class, the probabilities of selecting “not at all,” “several days,” “more than half the days,” or “nearly every day” across the nine depressive-symptom items. Based on their distinct response patterns, these three categories were designated as the “low depressive symptoms group”, “somatic-dominant depressive symptoms group,” and “severe-spectrum depressive symptoms group.”

The low depressive symptoms group (Class 3) was the largest cohort, accounting for 54.1% of the total sample (*n* = 178). Participants in this group most often selected “not at all” across all nine items, indicating the lowest depressive burden and minimal distress.

The somatic-dominant depressive symptoms group (Class 2) accounted for 22.5% of the sample (*n* = 74). Compared with the low depressive symptoms group, they were more likely to choose “several days” or “more than half the days” across items, particularly Items 3 (Sleep) and Items 4 (Fatigue). The probability of choosing “nearly every day” remained low across items, consistent with mild-to-moderate symptom burden.

The severe-spectrum depressive symptoms group (Class 1) accounted for 23.4% of the sample (*n* = 77). This group exhibited the most pronounced and frequent depressive symptoms. They were more likely than the other groups to select “More than half the days” or “Nearly every day” for most items, with cumulative probabilities exceeding 0.50 for many. These patterns indicate severe and frequent symptoms, identifying a high-risk cohort that warrants priority monitoring and intervention.

### 3.3 Comparisons of participant characteristics across the latent classes

Univariate analyses (Table 3) indicated significant differences across classes in age, occupation, comorbidities, self-rated health, ADL, and psychosocial factors (*p* < 0.05). Class 3 exhibited the most favorable profile with the highest rates of good self-rated health, ADL independence, and social participation. In contrast, Class 1 comprised the oldest residents (often ≥90 years) with the greatest physical dependency, multimorbidity, and social isolation. Class 2 showed intermediate characteristics. No significant differences were observed in sex, marital status, BMI, education, number of children, visit frequency, residence type, or length of stay.

**Table 3:** Comparison of resident characteristics across three latent classes of depressive symptoms (*n* = 329).

Variables	Class 1	Class 2	Class 3	Total	*p*
BMI *n* (%)					0.856
Underweight	20 (6.1)	17 (5.2)	39 (11.9)	76 (23.1)	
Normal	47 (14.3)	43 (13.1)	109 (33.1)	199 (60.5)	
Overweight	10 (3.0)	14 (4.3)	30 (9.1)	54 (16.4)	
Sex *n* (%)					0.279
Male	31 (9.4)	24 (7.3)	77 (23.4)	132 (40.1)	
Female	46 (14.0)	50 (15.2)	101 (30.7)	197 (59.9)	
Age group, years *n* (%)					0.002
64–74	8 (2.4)	7 (2.1)	30 (9.1)	45 (13.7)	
75–89	41 (12.5)	56 (17.0)	118 (35.9)	215 (65.3)	
≥90	28 (8.5)	11 (3.3)	30 (9.1)	69 (21)	
Education *n* (%)					0.376
Primary and below	23 (7.0)	29 (8.8)	56 (17.0)	108 (32.8)	
Middle School	21 (6.4)	13 (4.0)	33 (10.0)	67 (20.4)	
High School	14 (4.3)	19 (5.8)	49 (14.9)	82 (24.9)	
College and above	19 (5.8)	13 (4.0)	40 (12.2)	72 (21.9)	
Marital status *n* (%)					0.617
Married	34 (10.3)	27 (8.2)	70 (21.3)	131 (39.8)	
No spouse					
(Widowed/divorced/unmarried)	43 (13.1)	47 (14.3)	108 (32.8)	198 (60.2)	
Number of children *n* (%)					0.169
0	4 (1.2)	1 (0.3)	3 (1.7)	8 (2.4)	
1	7 (2.1)	8 (2.4)	33 (10.0)	48 (14.6)	
2	32 (9.7)	27 (8.2)	74 (22.5)	133 (40.4)	
3	22 (6.7)	23 (7.0)	49 (14.9)	94 (28.6)	
4 or more	12 (3.6)	15 (4.6)	19 (5.8)	46 (14.0)	
Frequency of visits in the previous 6 months *n* (%)					0.833
Almost every day	13 (4.0)	8 (2.4)	27 (8.2)	48 (14.6)	
At least once a week	40 (12.2)	43 (13.1)	101 (30.7)	184 (55.9)	
At least once a month	18 (5.5)	14 (4.3)	35 (10.6)	67 (20.4)	
At least once every 6 months	6 (1.8)	9 (2.7)	15 (4.6)	30 (9.1)	
Occupation before retirement *n* (%)					0.006
Party and government organization workers	2 (0.6)	0 (0)	10 (3.0)	12 (3.6)	
Workers in public organizations	35 (10.6)	20 (6.1)	53 (16.4)	108 (32.8)	
Enterprise personnel	8 (2.4)	25 (7.6)	50 (15.2)	83 (25.2)	
Free traders	2 (0.6)	6 (1.8)	3 (0.9)	11 (3.3)	
Laborers	13 (4.0)	10 (3.0)	26 (7.9)	49 (14.9)	
Farmers	13 (4.0)	11 (3.3)	25 (7.6)	49 (14.9)	
Soldiers	4 (1.2)	2 (0.6)	11 (3.3)	17 (5.2)	
Number of comorbidities *n* (%)					<0.001
0–1 types	12 (3.6)	9 (2.7)	63 (19.1)	84 (25.5)	
2–3 types	43 (13.1)	44 (13.4)	87 (26.4)	174 (52.9)	
≥4	22(6.7)	21(6.4)	28(8.5)	71(21.6)	
Self-rated health *n* (%)					<0.001
Very good	3 (0.9)	6 (1.8)	17 (5.2)	26 (7.9)	
Good	8 (2.4)	14 (4.3)	65 (19.8)	87 (26.4)	
Fair	23 (7.0)	20 (6.1)	59 (17.9)	102 (31)	
Poor	31 (9.4)	26 (7.9)	27 (8.2)	84 (25.5)	
Very poor	12 (3.6)	8 (2.4)	10 (3.0)	30 (9.1)	
Length of stay *n* (%)					0.288
≥3 months<7 months	21 (6.4)	18 (5.5)	55 (16.7)	94 (28.6)	
≥7 months<12 months	4 (1.2)	9 (2.7)	20 (6.1)	33 (10.0)	
1–3 years	31 (9.4)	24 (7.3)	46 (14.0)	101 (30.7)	
≥3 years	21 (6.4)	23 (7.0)	57 (17.3)	101 (30.7)	
Frequency of participation in group activities *n* (%)					<0.001
Regular	20 (6.1)	34 (10.3)	88 (26.7)	142 (43.2)	
Occasionally	22 (6.7)	16 (4.9)	50 (15.2)	88 (26.7)	
Never	35 (10.6)	24 (7.3)	40 (12.2)	99 (30.1)	
Type of residence *n* (%)					0.640
Living alone	18 (5.5)	26 (7.9)	60 (18.2)	104 (31.6)	
Living with spouse	27 (8.2)	20 (6.1)	55 (16.7)	102 (31.0)	
Living with other older people	32 (9.7)	28 (8.5)	63 (19.1)	123 (37.4)	
ADL *n* (%)					<0.001
Normal	18 (5.5)	24 (7.3)	112 (34.0)	154 (46.8)	
Mildly dependent	43 (13.1)	47 (14.3)	62 (18.8)	152 (46.2)	
Moderately dependent	16 (4.9)	3 (0.9)	4 (1.2)	23 (7.0)	
Social support level M ± SD					
Social support score	44.94 ± 8.778	49.19 ± 7.977	53.90 ± 8.194	50.74 ± 9.062	<0.001
Support from family	16.14 ± 2.941	17.15 ± 2.509	18.57 ± 2.529	17.68 ± 2.811	<0.001
Support from friend	14.48 ± 3.397	15.76 ± 2.988	17.48 ± 2.917	16.39 ± 3.294	<0.001
Support from a significant other	14.03 ± 2.585	15.09 ± 2.121	15.77 ± 1.985	15.21 ± 2.276	<0.001

Class 1: severe-spectrum depressive symptoms group; Class 2: somatic-dominant depressive symptoms group; Class 3: low depressive symptoms group, ADL, activities of daily living.

The results of the multivariable logistic regression analyses are presented in Table 4. Multinomial logistic regression was used to assess the clinical relevance of the classes identified by LCA. Perceived social support was significantly associated with class membership. Higher levels of support from family and friends were associated with lower odds of belonging to Class 1. Specifically, for every one-point increase in family support score, the odds of being in Class 1 rather than Class 2 decreased by approximately 15% (OR = 0.849, 95% CI: 0.784–0.919; *p* < 0.001). A similar association was observed for Class 1 versus Class 3 (OR = 0.864, 95% CI: 0.802–0.930; *p* < 0.001). Higher support from friends was also associated with lower odds of belonging to Class 1 (Class 1 vs. Class 2: OR = 0.890, 95% CI: 0.825–0.959; *p* = 0.002; Class 1 vs. Class 3: OR = 0.887, 95% CI: 0.827–0.952; *p* = 0.001). Overall, the observed associations between class membership and perceived social support supported the interpretability and clinical relevance of the identified classes. Support from a significant other was positively associated with Class 1 versus Class 2 in the fully adjusted model. Because this association appeared only in one pairwise contrast and was not consistent across other comparisons, it was interpreted cautiously and was not emphasized as a primary finding.

**Table 4:** Multinomial logistic regression analysis of factors associated with latent classes of depressive symptoms.

Variables	Class 1 vs. Class 2			Class 1 vs. Class 3			Class 2 vs. Class 3		
	OR	95% CI	*p*	OR	95% CI	*p*	OR	95% CI	*p*
Support from family	0.849	0.784–0.919	<0.001	0.864	0.802–0.930	<0.001	1.018	0.962–1.076	0.539
Support from friend	0.890	0.825–0.959	0.002	0.887	0.827–0.952	0.001	0.998	0.942–1.056	0.933
Support from a significant other	1.093	1.010–1.182	0.027	1.044	0.969–1.125	0.256	0.955	0.903–1.011	0.115
Age group, years									
64–74	0.398	0.099–1.606	0.195	0.287	0.086–0.954	0.042	0.721	0.223–2.335	0.585
75–89	0.288	0.114–0.724	0.008	0.370	0.161–0.852	0.020	1.286	0.556–2.974	0.556
≥90 (reference)	1	/	/	1	/	/	1	/	/
Number of comorbidities									
0–1 types	1.720	0.488–6.06	0.398	0.490	0.163–1.475	0.205	0.285	0.106–0.765	0.013
2–3 types	0.975	0.404–2.358	0.956	0.887	0.374–2.106	0.786	0.909	0.431–1.920	0.803
≥4 (reference)	1	/	/	1	/	/	1	/	/
Self-rated health									
Very good	0.270	0.037–1.984	0.198	0.198	0.030–1.309	0.093	0.734	0.174–3.090	0.673
Good	0.219	0.047–1.019	0.053	0.111	0.026–0.485	0.003	0.509	0.148–1.751	0.284
Fair	0.731	0.196–2.729	0.641	0.358	0.100–1.279	0.114	0.490	0.152–1.578	0.232
Poor	0.690	0.188–2.535	0.576	0.948	0.258–3.490	0.936	1.375	0.411–4.601	0.605
Very poor (reference)	1	/	/	1	/	/	1	/	/
Frequency of participation in group activities									
Regular	0.460	0.188–1.122	0.088	0.310	0.132–0.725	0.007	0.674	0.327–1.388	0.284
Occasionally	0.986	0.376–2.583	0.977	0.688	0.287–1.647	0.401	0.698	0.304–1.602	0.396
Never (reference)	1	/	/	1	/	/	1	/	/
ADL									
Normal	0.223	0.043–1.162	0.075	0.069	0.015–0.312	0.001	0.309	0.055–1.734	0.182
Mildly dependent	0.242	0.051–1.142	0.073	0.247	0.058–1.056	0.059	1.024	0.188–5.572	0.978
Moderately dependent (reference)	1	/	/	1	/	/	1	/	/

A *p*-value <0.05 was considered statistically significant. In each pairwise comparison, the latter class was used as the reference group (Class 2 for Class 1 vs. Class 2; Class 3 for Class 1 vs. Class 3 and Class 2 vs. Class 3).

## 4 Discussion

We used LCA to identify three depressive-symptom profiles among older adults living in LTC homes. Our findings suggest that depressive symptoms in this population are heterogeneous and may be represented by clinically interpretable symptom patterns, rather than a wholly uniform severity continuum. From a clinical perspective, complementing severity-based screening with symptom-pattern-informed stratification may help prioritize residents for targeted assessment and tailored care (e.g., sleep- and fatigue-focused support versus broader psychosocial intervention).

The depressive symptom subgroups identified in this study were broadly comparable to those reported in previous LTC studies from other countries. In particular, the low depressive symptoms group and the severe-spectrum depressive symptoms group were broadly consistent with patterns reported in previous LTC studies (11). By contrast, the intermediate group in our study was characterized more by sleep disturbance and fatigue than by a clearly emotional presentation. This suggests that, although some broad patterns of depressive symptom heterogeneity may recur across studies, the specific composition of intermediate subgroups may be more sensitive to institutional context and residents’ underlying health burden (45, 46).

The severe-spectrum depressive symptoms group was characterized by advanced age, multiple chronic conditions, greater ADL limitations, poorer self-rated health, and lower social support. Overall, this profile is consistent with prior evidence that depressive symptoms in LTC home residents are associated with multimorbidity, functional limitations, and psychosocial disadvantages (42, 43). Compared with other groups, they were disadvantaged across physical, psychological, and social domains. Specifically, multimorbidity (which increases with older age) is associated with greater body pain and discomfort (47). Pain and functional limitations may, in turn, reduce residents’ ability to take part in activities within LTC homes (48–50), potentially increasing social withdrawal. Reduced participation and withdrawal have been linked to loneliness and lower perceived support (51). Taken together, these factors may co-occur one another across physical, functional, and psychosocial domains (48–50). These differences may have implications for LTC management and care. First, managers of LTC homes may consider enhanced psychological screening for very old residents and those with multimorbidity. Second, care providers may help inform psychological support into routine care for residents with functional limitations. Additionally, LTC homes should mitigate isolation by expanding group activities and creating more opportunities for social interaction. Taken together, these measures may be relevant to depressive symptom burden and may inform care assessment or support planning.

The somatic-dominant depressive symptoms group had a moderate overall symptom burden but was characterized by relatively higher endorsement of sleep disturbance and fatigue, rather than uniformly high endorsement across all PHQ-9 items. Similar subgroups dominated by sleep disturbance and fatigue have been reported previously (52). This pattern should be interpreted cautiously. In LTC residents, sleep disturbance and fatigue may reflect depressive symptoms, and one possible interpretation is that emotional distress may be expressed through somatic complaints in some cultural contexts (53). However, these symptoms may also arise from chronic disease burden, frailty, pain, medication effects, sleep disorders, or other geriatric conditions, and the present data cannot determine whether they were primarily depression-related or attributable to physical illness. Moreover, sleep disturbance and fatigue have been associated with depressive symptoms, poorer sleep-related outcomes, reduced daytime functioning, social withdrawal, and functional decline among older adults (46, 54, 55). Accordingly, the somatic-dominant class should be understood as an empirically derived PHQ-9 symptom-response pattern, rather than a definitive depressive subtype. Clinically, this pattern may help identify residents who require further assessment of both psychological symptoms and physical health conditions.

The low depressive symptoms group, the largest cohort, showed the lowest overall depressive symptoms burden. This group had optimal ADL function, the highest engagement in group activities, and the strongest social support network. Adequate physical function underpins independent living in older adults and is a prerequisite for social participation. Regular group and social participation is associated with more favorable emotional well-being and are a primary pathway to building peer relationships and obtaining social support (56, 57). High social integration and perceived support serve as key coping resources that is linked to lower monotony and loneliness in LTC settings.

Our observed rates of depressive symptoms exceeded those reported in other LTC settings in China (58). In addition to differences in measurement methods and case composition, data collection primarily took place during the summer months, when some LTC homes may have modified or reduced group activities in response to heat-related safety concerns (59). While we did not directly measure activity restrictions or seasonal exposure patterns, others have reported on the association between short-term reductions in social engagement and negative emotional outcomes among older adults (60, 61). In LTC homes, daily social contact provides essential psychological support amid routines. Therefore, even brief reductions in social engagement may be related to loneliness and social isolation, with adverse emotional consequences (62, 63). Short-term isolation may also disrupt routines and diminish the positive effect and comfort derived from group activities. These changes have been linked to increasing the risk of higher depressive symptom burden (60, 64). Accordingly, short-term fluctuations in social interaction warrant attention, not only prolonged absence.

The observed association between depressive symptom profiles and perceived social support adds to the clinical relevance of the identified profiles. Residents in the severe-spectrum depressive symptoms group reported lower social support scores than those in the other groups. This finding is consistent with previous evidence showing that social support is closely associated with late-life depressive symptoms (65, 66). Higher support from family or friends was associated with lower odds of membership in the severe-spectrum depressive symptoms group. Given the cross-sectional design and the use of a perceived support scale, the direction of the association cannot be established. Depressive symptoms may also influence how residents perceive the availability or adequacy of social support, and reciprocal relationships cannot be ruled out. A key finding of this study is the discrepancy between structural and functional support. While structural indicators (e.g., number of children, visit frequency) did not differ significantly across classes, perceived social support scores varied sharply. This suggests that for LTC residents, the quality of support—the subjective experience of feeling understood and valued—may be more critical than the quantity of contact (67, 68). Transitioning to a LTC home can reshape family involvement from direct hands-on caregiving toward maintaining emotional connection, making residents more sensitive to the perceived quality of that connection (69). LTC providers should build multilevel social support networks when implementing mental-health interventions for residents (50). LTC homes should encourage family visits and leverage digital tools (e.g., video calls) to sustain accessible family support (70). At the institutional level, fostering residents and staff support, as well as offering meaningful shared activities, can help promote friendships and reduce loneliness and isolation (71).

### 4.1 Strengths and limitations

This study has several strengths. We adopted a person-centered approach and used LCA to identify depressive-symptom subgroups among residents of LTC homes in China. This approach yielded a clinically interpretable classification model that can underpin stratified management and targeted interventions. Additionally, questionnaires were interviewer-administered: trained data collectors read items aloud and recorded residents’ responses. This method is recommended for older residents in nursing homes because sensory, physical, or cognitive impairments may hinder independent self-completion (72).

This study also has several limitations. First, the sample was drawn from a limited number of LTC homes in Qingdao, which may restrict the generalizability of the findings to broader geographic regions, rural or lower-resource settings, or institutions with different care levels. Institutional characteristics, including staffing, activity provision, medical service access, and resident case-mix, may also influence depressive symptom patterns but were not systematically examined. Second, residents with severe cognitive impairment were not included because they could not reliably complete the PHQ-9 interview and other self-report measures. Although this exclusion was necessary to ensure response validity, it may have influenced the composition of the latent classes and may have underrepresented severe or dementia-related depressive symptom patterns (73).

Third, several analysis-related limitations should be acknowledged. Residual dependence was observed among some PHQ-9 item pairs, and binary recoding showed lower agreement with the original classification; therefore, the identified classes should be interpreted as empirically derived symptom-response patterns rather than definitive clinical subtypes. In addition, measurement invariance and differential item functioning across sex or age groups were not examined because subgroup sample sizes were limited for stable multiple-group LCA. The multinomial regression results should also be interpreted as exploratory because the subgroup sizes were modest, limiting the ability to assess interaction effects or estimate more complex models. Future studies with larger samples should examine whether the identified class structure is invariant across key demographic groups. Fourth, all measures were based on self-reports, which may introduce recall bias and social desirability bias, particularly within a cultural context where mental health stigma persists (74). Fifth, we did not collect detailed information on frailty, chronic pain, diagnosed sleep disorders, antidepressant or sedative medication use, or cognitive impairment severity below the exclusion threshold. Therefore, we could not fully distinguish depression-related somatic symptoms from symptoms attributable to physical illness, medication effects, sleep problems, or cognitive impairment. Future studies should include more detailed geriatric assessments to clarify this issue. Finally, the cross-sectional design precludes causal inferences. Despite adjustment for multiple confounders, residual confounding from unmeasured factors such as socioeconomic status and duration of institutionalization cannot be ruled out. Therefore, larger, multi-site longitudinal studies are needed to examine the stability and generalizability of these class profiles and clarify temporal pathways.

## 5 Conclusion

This study suggests that depressive symptoms among older adults living in LTC homes are heterogeneous and can be described by clinically interpretable symptom patterns identified through LCA (18–20). Three subgroups were identified: a low depressive symptoms group, a somatic-dominant depressive symptoms group, and a severe-spectrum depressive symptoms group. Overall, our findings are consistent with the multifactorial nature of depressive symptoms, in which symptom burden tends to co-occur with medical, functional, and psychosocial vulnerabilities rather than reflecting a single factor (75). Notably, lower perceived support from family and friends was associated with membership in the severe-spectrum group, suggesting that perceived social support may be relevant to depressive symptom differentiation in LTC residents (76). From a care perspective, symptom-pattern–informed stratification may help prioritize residents who are oldest-old, physically dependent, and socially isolated for targeted assessment and tailored interventions. Multilevel strategies that address both functional health and social connectedness may offer a practical approach to supporting mental health in LTC settings (8).

## Data Availability

The datasets presented in this article are not readily available because the raw data from this study contains information that could identify individual patients and therefore cannot be made public or disclosed to third parties. All data collected during the study has been anonymized in accordance with ethical requirements and is used only within the research team under controlled conditions to ensure the privacy and security of the participants. Requests to access the datasets should be directed to Yuting Song, songyuting@qdu.edu.cn.
